# Supplementary material for: Analyzing microbial evolution through gene and genome phylogenies
Source: Biostatistics. 2023 Oct 28;25(3):786–800. doi: 10.1093/biostatistics/kxad025 (PMC11247178; doi:10.1093/biostatistics/kxad025)
Supplement: kxad025_Supplementary_Data [file biosts-23053-File001.pdf › biosts-23053-File001.pdf]

NCBI Multiple Sequence Alignment Viewer, Version 1.22.0

Alignment

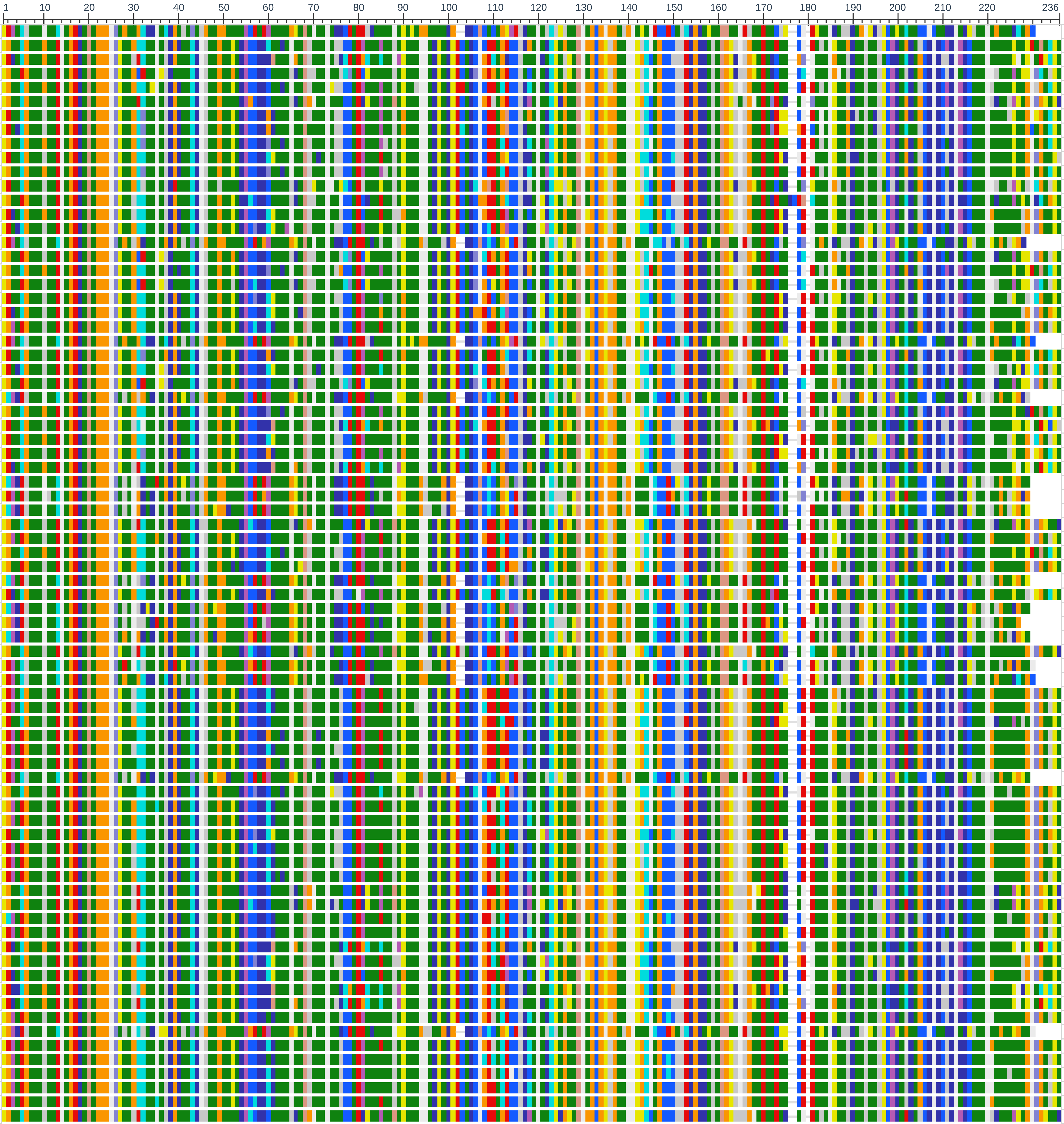

Sequence ID	Start	End	Organism
GCA_000025925.1(+)	1	225	
GCA_000144405.1(+)	1	233	
GCA_000157935.1(+)	1	233	
GCA_000177075.1(+)	1	233	
GCA_000191065.1(+)	1	233	
GCA_000218235.1(+)	1	233	
GCA_000220235.1(+)	1	233	
GCA_000220255.1(+)	1	233	
GCA_000257925.1(+)	1	233	
GCA_000373185.1(+)	1	233	
GCA_000377625.1(+)	1	233	
GCA_000378085.1(+)	1	233	
GCA_000379965.1(+)	1	235	
GCA_000382385.1(+)	1	233	
GCA_000424185.1(+)	1	233	
GCA_000426565.1(+)	1	223	
GCA_000455445.1(+)	1	233	
GCA_000467895.1(+)	1	233	
GCA_000479005.1(+)	1	233	
GCA_000518545.1(+)	1	233	
GCA_000599605.1(+)	1	233	
GCA_000621725.1(+)	1	233	
GCA_000687715.1(+)	1	225	
GCA_000688375.1(+)	1	233	
GCA_000758925.1(+)	1	233	
GCA_000762405.1(+)	1	233	
GCA_000834015.1(+)	1	224	
GCA_001262015.1(+)	1	233	
GCA_001275135.1(+)	1	233	
GCA_001552765.1(+)	1	233	
GCA_001953955.1(+)	1	233	
GCA_002224675.1(+)	1	233	
GCA_002251295.1(+)	1	224	
GCA_002251365.1(+)	1	224	
GCA_002265625.1(+)	1	224	
GCA_002353825.1(+)	1	233	
GCA_002394365.1(+)	1	233	
GCA_003609775.1(+)	1	233	
GCA_003977605.1(+)	1	233	
GCA_004535825.1(+)	1	224	
GCA_008016795.1(+)	1	233	
GCA_009495355.1(+)	1	224	
GCA_013166515.1(+)	1	222	
GCA_013166545.1(+)	1	224	
GCA_013333935.1(+)	1	233	
GCA_900100635.1(+)	1	225	
GCA_900107775.1(+)	1	225	
GCA_900109055.1(+)	1	233	
GCA_900110895.1(+)	1	233	
GCA_900113305.1(+)	1	233	
GCA_900119285.1(+)	1	233	
GCA_900142525.1(+)	1	233	
GCA_900199555.1(+)	1	233	
GCA_900290275.1(+)	1	224	
GCA_900314455.1(+)	1	233	
GCA_900314715.1(+)	1	233	
GCA_900314755.1(+)	1	233	
GCA_900314945.1(+)	1	233	
GCA_900314995.1(+)	1	233	
GCA_900315525.1(+)	1	233	
GCA_900315565.1(+)	1	233	
GCA_900315635.1(+)	1	233	
GCA_900315835.1(+)	1	233	
GCA_900316295.1(+)	1	233	
GCA_900318585.1(+)	1	233	
GCA_900544825.1(+)	1	233	
GCA_900546345.1(+)	1	233	
GCA_900637655.1(+)	1	233	
GCA_900771975.1(+)	1	233	
GCA_902167755.1(+)	1	233	
GCA_902760345.1(+)	1	233	
GCA_902762565.1(+)	1	225	
GCA_902769705.1(+)	1	233	
GCA_902770195.1(+)	1	233	
GCA_902774925.1(+)	1	233	
GCA_902776195.1(+)	1	233	
GCA_902788605.1(+)	1	233	
GCA_902792635.1(+)	1	233	
